# COVID-19 positive woman presented with major fetal congenital anomalies: A case report with literature review

**DOI:** 10.1097/MD.0000000000039504

**Published:** 2024-09-06

**Authors:** Sezgi Güllü Erciyestepe, Özlem Pata

**Affiliations:** a Department of Obstetrics and Gynecology, Acibadem Health Group Bakirkoy Hospital, Istanbul, Turkey; b Department of Perinatology, Acibadem Health Group Bakirkoy Hospital, Istanbul, Turkey.

**Keywords:** coronavirus, COVID-19, first-trimester, pregnancy, respiratory

## Abstract

**Rationale:**

Pregnancy is a special term in life with physiological changes in both cardiorespiratory and immune systems; that is why severe acute respiratory syndrome coronavirus 2 infection in pregnancy may result in an altered response. With this, we present a case report of a young pregnant lady who was exposed to severe acute respiratory syndrome coronavirus 2 infection just before pregnancy and ended up with an affected fetus. The impact of coronavirus disease 2019 (COVID-19) exposure on neonatal outcomes has not yet been fully evaluated; by this article, we aim to find if COVID-19 exposure is linked to congenital anomalies.

**Patient concerns:**

A 25-year-old woman who has no history of genetic or chronic diseases applied to our clinic for routine control of pregnancy. She does not have a consanguineous marriage or any other potential risk factors for pregnancy.

**Diagnoses and interventions:**

She had a history of COVID-19 polymerase chain reaction positivity 2 days before the first day of the last menstruation period and hospitalization for 7 days. After 7 days of treatment with favipiravir and levofloxacin, enoxaparin sodium, famotidine, paracetamol, budesonide, dornaz alfa, and vitamin C; her general situation gets better, and discharged from the hospital on the seventh day of hospitalization without any further treatment prescription.

**Outcomes:**

During her routine controls for pregnancy at first-trimester evaluation ultrasonography; there was right forearm aplasia and deformities at both feet and legs.

**Lessons:**

In the literature, there is conflicting evidence about the impact of COVID-19 in pregnancy especially if the patient is confronted with the virus in the first trimester. Despite the increasing number of published studies on COVID-19 in pregnancy, there are insufficient good quality unbiased studies about the issue. Risk factors for COVID-19 overlap with the risk factors for pregnancy complications and the risk factors of the treatment prescribed. The impact of COVID-19 exposure on neonatal outcomes has not yet been fully evaluated; in this article, we aim to find if COVID-19 exposure is linked to congenital anomalies. Further research is needed to ascertain neonatal outcomes.

## 1. Introduction

Pregnancy is a special term in life with physiological changes in both cardiorespiratory and immune systems; that is why severe acute respiratory syndrome coronavirus 2 (SARS-CoV2) infection in pregnancy may result in an altered response. Fetuses may be exposed to infection during critical periods of development; especially first-trimester exposure is miscellaneous. There are mostly case reports of second and third-trimester pregnancies with SARS-CoV2 infection in the literature and meta-analysis, and systemic reviews done based on those case reports. With this, we present a case report of a young pregnant lady who was exposed to SARS-CoV2 infection just before pregnancy and ended up with an affected fetus.

## 2. Case presentation

### 2.1. Patient information

Twenty-five-year-old woman who has no history of genetic or chronic diseases applied to our clinic for routine control of pregnancy. She does not have a consanguineous marriage or any other potential risk factors for pregnancy. She had a history of Coronavirus Disease 2019 (COVID-19) PCR positivity 2 days before the first day of the last menstruation period and hospitalization for 7 days. At her admission to the hospital with weakness of muscles and flu-like symptoms (cough, dizziness, and headache), physical examination findings were at normal range rather than increased respiratory rate: fever measured as 37 **°**C, oxygen saturation was 98%, and blood pressure was 110/70 mm Hg. Covid PCR test was positive and computed tomography showed an atypical viral pneumonia-like frosted glass image at the posterobasal segment at the left lung lower lobe. Blood tests resulted in thrombocytopenia (131 × 10^3^/µL) and C reactive protein (CRP) was 7.2 mg/dL (0–0.5 mg/dL). Her beta human chorionic gonadotropin (beta hCG) blood test result was negative. After hospitalization during her follow-up when fever was measured as 38 **°**C, blood culture tests were taken and resulted negative. During her follow-up the results of leucocyte, thrombocyte, and CRP were 3.48 × 10^3^/µL, 116 × 10^3^/µL, and 6.8 mg/dL, respectively. After 7 days of treatment with favipiravir and levofloxacin, enoxaparin sodium, famotidine, paracetamol, budesonide, dornaz alfa, and vitamin C; her general situation gets better, and discharged from the hospital on 7th day of hospitalization without any further treatment prescription. On her discharge day, leucocyte, thrombocyte, and CRP tests resulted in 4.5 × 10^3^/µL, 114 × 10^3^/µL, and 3.2 mg/dL, respectively. One week after hospitalization COVID PCR test resulted as negative and beta hCG resulted positive 2 weeks after hospital discharge.

### 2.2. Clinical findings and diagnostic assessment

**Figure 1.**
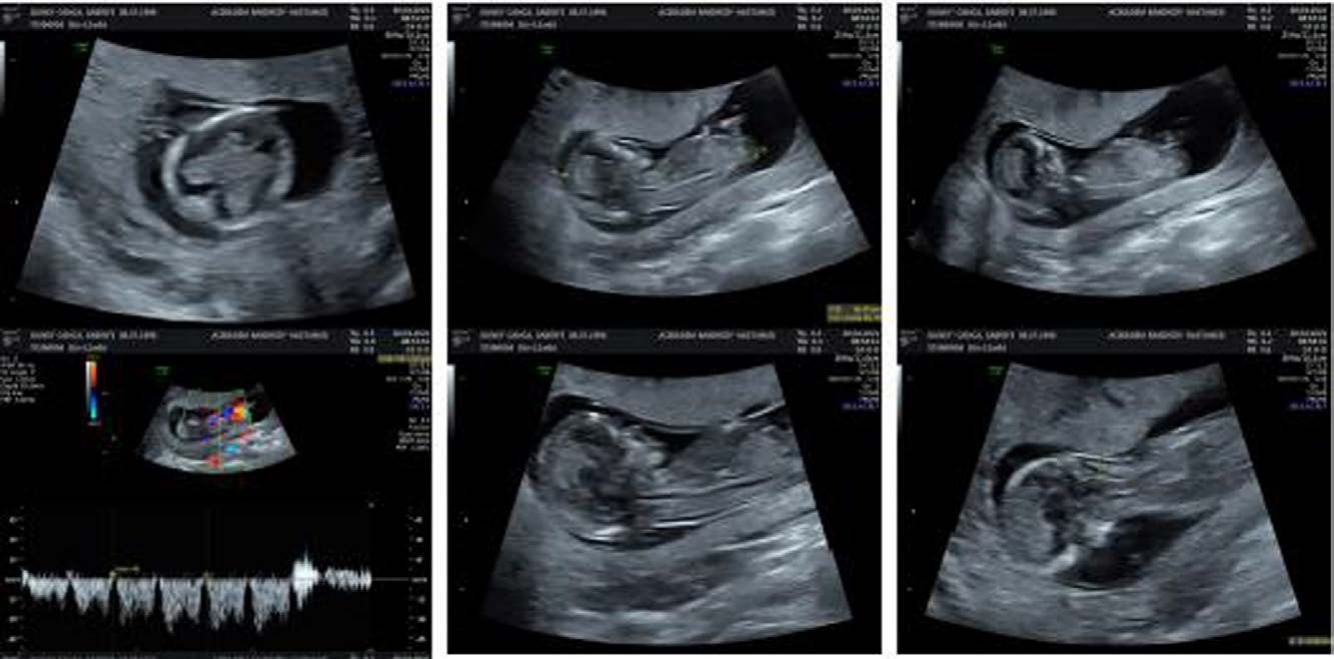
First-trimester ultrasonographic images.

**Figure 2.**
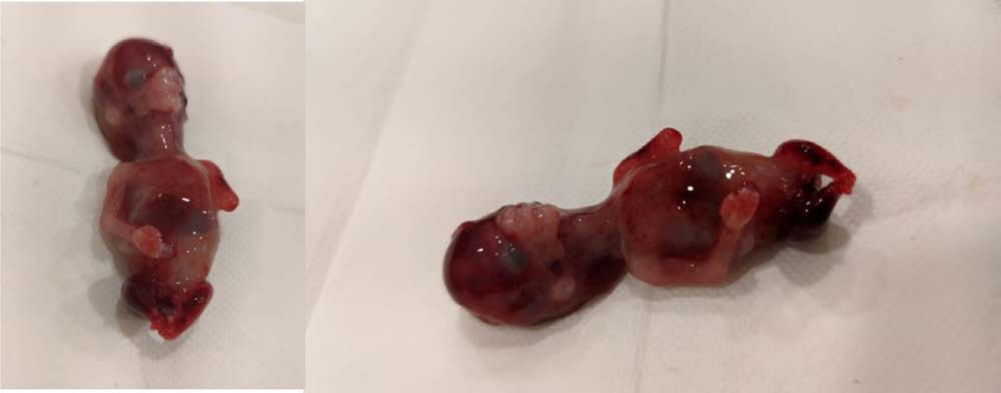
Fetus with forearm aplasia and sirenomelia.

During her routine controls for pregnancy at first-trimester evaluation ultrasonography; no anomalies related to the head, neck, vertebra, thorax, heart, abdominal wall, stomach, kidneys, or bladder were seen. Nazal bone was also seen and there was not any reverse ductal current in Doppler ultrasonography. There was right forearm aplasia and deformities at both feet and legs. Other than isolated anomalies at extremities, all fetal biometry measurements were normal, the amniotic fluid index was at normal range and the posteriorly located placenta seems natural. First-trimester ultrasonographic images can be seen in Figure 1. Chorionic villus sampling and DNA analysis: whole exome sequencing was done. Whole exome sequencing resulted as negative and there was no variant gene to explain sirenomelia. Sirenomelia is characterized by complete or incomplete fused lower extremities, renal agenesis, oligohydramnios, absent urinary tract and external genitalia, single umbilical arteries, and imperforate anus.^[1]^ It is a very rare lethal congenital anomaly; a retrospective cohort study done in North and South America, Europe, China, and Australia showed global incidence as 0.98/100,000.^[1]^ Perinatology offered the option of termination of pregnancy to the patient. The pregnancy terminated on the 13th of the gestational week and was sent to a genetic laboratory for further analysis. In Figure 2, images of the fetus with sirenomelia and forearm aplasia can be seen.

### 2.3. Follow-up and outcomes

Today, she already got pregnant for the second time and there were not any anticipated problems during her pregnancy follow-ups and she gave a healthy birth with a cesarean operation.

## 3. Discussion

As the fetal organs develop during the first trimester of the pregnancy, maternal infections at this stage may be more dangerous compared to later gestational ages.^[2]^ This case has great significance since the patient has COVID-19 disease and treatment before beta hCG test positivity. That comes with a question does medicine or COVID-19 disease affect ovum quality and cause fetal malformation before even implantation? Literature has limited case reports about confronting the virus in the first trimester: and most of them report miscarriage rather than any malformation. Setti et al compared infertile couples’ artificial reproductive techniques outcomes between the pre-COVID and COVID-19 periods and results show no significant differences in terms of pregnancies per cycle (*P* = .237), biochemical pregnancy rates (*P* = .089), ectopic pregnancy (*P* = .594), spontaneous miscarriage (*P* = .487), and intrauterine ongoing pregnancies (*P* = .569).^[3]^ La Cour Freiesleben et al in their cohort study showed that SARS-CoV2 infection does not have a significant effect on nuchal translucency thickness measured at the first-trimester scan and does not have a significant increase in pregnancy loss.^[4]^

In another study by Liu et al, there were no statistical differences in terms of percentages of cesarean, premature rupture of membranes, pregnancy with hypothyroidism, placental abnormality, abnormal amniotic fluid volume, pregnancy with thrombocytopenia, pregnancy with anemia (*P* > .05) between COVID-19 infected and the control groups; the rates of gestational diabetes mellitus was higher in COVID-19 infected group (*P* < .05).^[5]^

In the literature, most of the reports are about 2nd and 3rd trimester COVID-19 positivity. When the patient confronts the virus in the second trimester; preterm birth or intrauterine growth restriction are the main outcomes with the increased occurrence and when the patient confronts the virus in the third trimester; there are cases of fetal deaths. The authors mainly emphasize the fact of intense placental inflammatory reaction as a direct effect of the disease.^[6]^

Rosen et al in their prospective cohort study with 55 pregnant women who had COVID-19 disease in the first and second trimesters; major anomalies in 2 fetuses were seen. In 1 case where the mother was infected with SARS-CoV2 at 8 weeks of gestation; anhydramnios and small echogenic kidneys were present and fetal blood sampling revealed no genetic abnormalities on chromosomal microarray testing. In another case, in a mother with mild symptomatic COVID-19 disease at the 15th gestational week, an isolated finding of unilateral fetal cataract was detected.^[7]^ With this, although it is shown that there are some abnormalities related to COVID-19; there is not any case report that shows the relation of COVID-19 with sirenomelia in the literature still.

For the reason of fetal malformation the medicine given should also be considered. In our case, 7 days of treatment with favipiravir (category X), levofloxacin (category C), enoxaparin sodium (category B), famotidine (category B), paracetamol (category B), budesonide (category B), dornaz alfa (category B), and vitamin C, were given to the patient and stopped 1 week before beta hCG positivity. In our country according to the health ministry data; more than 103,000 humans died due to COVID-19. COVID-19 causes morbidity as well as mortality. Our case was diagnosed with COVID-19 in June 2022 and the routine treatment of COVID-19 in our country at that date was the treatment administered to our patient. Now, the treatment approaches differ between the health centers. Favipiravir, an antiviral drug, is an RNA polymerase inhibitor and has been used for the treatment of COVID-19 disease.^[8]^ Favipiravir use is contraindicated during pregnancy; the drug has a high risk for teratogenicity and embryotoxicity. A retrospective study done by Tirmikçioğlu et al with 29 women who were exposed to favipiravir during pregnancy resulted in 25 live births, 1 foramen ovale, and 2 preterm deliveries. The study indicated that 29 exposures occurred during the contraindicated time (from 7 days before conception and/or during the pregnancy). One of 25 live birth infants showed some minor congenital anomalies (patent foramen ovale); other than that there were no major congenital anomalies.^[9]^ In another study done by Ethem et al, favipiravir was indicated as not likely to be a teratogen since no major congenital anomalies were found in a retrospective study that covered up 1 year of admissions.^[10]^ In our case, the question is whether the malformation is related to COVID-19 infection or favipiravir usage. Since there are more reported congenital malformations caused by COVID-19 infection rather than favipiravir; we as the authors think that the sirenomelia seen in this fetus is most likely due to the result of COVID-19 infection. Although we as the authors linked the malformations to COVID-19; in the reproductive period the treatment prescribed should be reconsidered by teratogenicity as well by considering advantages and disadvantages.

In the literature, there is conflicting evidence about the impact of COVID-19 in pregnancy especially if the patient is confronted with the virus in the first trimester. Despite the increasing number of published studies on COVID-19 in pregnancy, there are insufficient good quality unbiased studies about the issue. Risk factors for COVID-19 overlap with the risk factors for pregnancy complications and the risk factors of the treatment prescribed. The impact of COVID-19 exposure on neonatal outcomes has not yet been fully evaluated; by this article, we aim to find if COVID-19 exposure is linked to congenital anomalies. Further research is needed to ascertain neonatal outcomes.

## Author contributions

**Conceptualization:** Erciyestepe Sezgi Güllü, Pata Özlem.

**Data curation:** Erciyestepe Sezgi Güllü, Pata Özlem.

**Resources:** Erciyestepe Sezgi Güllü.

**Supervision:** Pata Özlem.

**Writing – original draft:** Erciyestepe Sezgi Güllü.

**Writing – review & editing:** Erciyestepe Sezgi Güllü.

## References

[1] Orioli IM Amar E Artega-Vazques J. Sirenomelia: an epidemiologic study in a large dataset from the International Clearinghouse of Birth Defects Surveillance and Research, and literature review. Am J Med Genet C Semin Med Genet. 2011;157c:358–73. 22002878 10.1002/ajmg.c.30324 PMC4492125

[2] Alvarado MG Schwartz DA. Zika virus infection in pregnancy, microcephaly, and maternal and fetal health: what we think, what we know, and what we think we know. Arch Pathol Lab Med. 2017;141:26–32. 27636525 10.5858/arpa.2016-0382-RA

[3] Setti PEL Cirillo F Immediata V. First-trimester pregnancy outcomes in a large IVF center from the Lombardy County (Italy) during the peak COVID-19 pandemic. Sci Rep. 2021;11:16529. 34400730 10.1038/s41598-021-96134-9 PMC8368203

[4] la Cour Freiesleben N Egerup P Hviid KV. SARS-CoV-2 in first trimester pregnancy: a cohort study. Hum Reprod. 2021;36:40–7. 33145598 10.1093/humrep/deaa311 PMC7665455

[5] Liu Y Dai M Tang S. Effect of initial COVID-19 outbreak during first trimester on pregnancy outcome in Wuxi, China. BMC Pregnancy Childbirth. 2022;22:54. 35062910 10.1186/s12884-022-04395-7 PMC8778492

[6] Richtmann R Torloni MR Oyamada Otani AR. Fetal deaths in pregnancies with SARS-CoV-2 infection in Brazil: a case series. Case Rep Womens Health. 2020;27:e00243. 32704477 10.1016/j.crwh.2020.e00243 PMC7354271

[7] Rosen H Bart Y Zlatkin R. Fetal and perinatal outcome following first and second trimester COVID-19 infection: evidence from a prospective cohort study. J Clin Med. 2021;10:2152. 34065646 10.3390/jcm10102152 PMC8156528

[8] Joshi S Parkar J Ansari A. Role of favipiravir in the treatment of COVID-19. Int J Infect Dis. 2021;102:501–8. 33130203 10.1016/j.ijid.2020.10.069 PMC7831863

[9] Tirmikçioğlu Z. Favipiravir exposure and pregnancy outcome of COVID-19 patients. Eur J Obstet Gynecol Reprod Biol. 2022;268:110–5. 34902747 10.1016/j.ejogrb.2021.12.001 PMC8647391

[10] Ertem O Guner O Incir C Kalkan S Gelal A. The outcomes of favipiravir exposure in pregnancy: a case series. Arch Gynecol Obstet. 2023;307:1385–95. 35622152 10.1007/s00404-022-06615-z PMC9136192

